# Potential of mucoadhesive nanocapsules in drug release and toxicology in zebrafish

**DOI:** 10.1371/journal.pone.0238823

**Published:** 2020-09-24

**Authors:** Ives Charlie-Silva, Natália Martins Feitosa, Juliana Moreira Mendonça Gomes, Daniela Chemim de Melo Hoyos, Cristiano Campos Mattioli, Silas Fernandes Eto, Dayanne Carla Fernandes, Marco Antonio de Andrade Belo, Juliana de Oliveira Silva, André Luis Branco de Barros, Jose Dias Corrêa Junior, Gustavo Batista de Menezes, Hirla Costa Silva Fukushima, Tássia Flávia Dias Castro, Ricardo Carneiro Borra, Felipe Pierezan, Nathalie Ferreira Silva de Melo, Leonardo Fernandes Fraceto

**Affiliations:** 1 Department of Pharmacology at University of São Paulo-ICB/USP, São Paulo-SP, Brazil; 2 Laboratório Integrado de Biociências Translacionais (LIBT), Instituto de Biodiversidade e Sustentabilidade (NUPEM), Universidade Federal do Rio de Janeiro (UFRJ)- Macaé, RJ, Brazil; 3 Department of Morphology, ICB-UFMG, Belo Horizonte-MG, Brazil; 4 Aquiculture Laboratory (Laqua), UFMG, Belo Horizonte-MG, Brazil; 5 Department of Preventive Veterinary Medicine, São Paulo State University, Jaboticabal-SP, Brazil; 6 Department of Postgraduate in Health Sciences—PROCISA, Federal University of Roraima (UFRR), Boa Vista, Brazil; 7 Institute of Chemistry, São Paulo State University (Unesp), Araraquara, São Paulo, Brazil; 8 Laboratory of Animal Pharmacology and Toxicology, Brasil University, Descalvado/SP, Brazil; 9 Department of Clinical and Toxicological Analyses, Faculty of Pharmacy-UFMG, Belo Horizonte-MG, Brazil; 10 Laboratory of Applied Immunology, Federal University of São Carlos, São Carlos-SP, Brazil; 11 School of Veterinary Medicine, Department of Clinic and Veterinary Surgery, UFMG, Belo Horizonte-MG, Brazil; 12 Faculty of Medicine São Leopoldo Mandic, Araras-SP, Brazil; 13 Institute of Science and Technology of Sorocaba, São Paulo State University, Sorocaba-SP, Brazil; VIT University, INDIA

## Abstract

Mucoadhesive polymeric nanocapsules have attracted interest of researchers from different fields from natural sciences because of their ability to interact with the mucosa and increase drug permeation. Anesthesia by immersion causes absorption through the skin and gills of fish, so it is important to evaluate the exposure of these organs to drug nanosystems. Benzocaine (BENZ) is one of the most popular anesthetic agents used in fish anesthesia, but it has drawbacks because of its low bioavailability, resulting in weak absorption after immersion. Here we describe method developed for preparing and characterizing chitosan-coated PLGA mucoadhesive nanoparticles containing BENZ (NPMAs) for zebrafish immersion anesthesia. We determined the lowest effective concentration, characterized the interaction of the mucoadhesive system with fish, measured the anesthetic efficacy, and evaluated possible toxic effects in embryos and adults exposed to the nanoformulations. This study opens perspectives for using nanoformulations prepared with BENZ in aquaculture, allowing reduction of dosage as well as promoting more effective anesthesia and improved interaction with the mucoadhesive system of fish.

## 1. Introduction

Despite bright outlooks for the future of nanotechnology, there is increasing concern of human and animal exposure to some types of engineered nanoparticles [1]. Nanoparticle-based drug delivery systems (NDDS) have been developed to prolong and optimize drug administration in relation to conventional formulations. NDDS show improved pharmacokinetics and bioavailability of drugs and reduced toxicity by their accumulation at the target site. They also enhance aqueous solubility and stability of various therapeutic agents as well as minimize tissue hypersensitivity reactions [2, 3]. Recently, mucoadhesive nanoparticles have gained attention for mucosal drug release [4, 5]. In this respect, chitosan-coated nanoparticles are surface-modified nanosystems with the ability to interact electrostatically with the mucosa [6] to overcome steric inhibition of mucin fibers [7] and enhance permeation of drugs due to the reorganization of intercellular junctions of the mucosa [8]. The composition of the fish mucus layer is like the mammal mucus layer, including the presence of mucin [9]. Therefore, these mucosal properties can be exploited for controlled release. In Accordance with Oberdörster et al. [1], there is concern of environmental contamination with the use of nanosystems and associated effects on ecosystems, which could have significant societal implications. In addition, there is a biological risk of animals exposed to such nanotechnology.

Several drugs have been applied in fish anesthetic procedures in order to reduce stress and damage to these organisms [10, 11]. There are few reports in the literature on residue concentrations of drugs that are administered to fish by bath immersion. Moreover, little is known about the impact of these drugs on the environment. Among the most used pharmaceuticals in aquaculture is benzocaine that is extensively used worldwide for the immobilization of fish during the handling or any other procedure that needs deep anesthesia [12, 13].

Benzocaine (BENZ), the ethyl ester of p-aminobenzoic acid, is a local anesthetic commonly used in human medicine and one of the most used for fish anesthesia [10, 14, 15]. Its anesthetic effects are desirable for teleost fish due to fast induction, short half-life, and rapid recovery time [16]. For these reasons, BENZ has been extensively used in fish production [17]. Despite the efficacy in fish, BENZ presents low bioavailability, resulting in weak and erratic absorption after immersion [18, 19]. The complex pharmacological profile of BENZ in fish remains poorly understood. To overcome these problems and improve anesthetic effects, release systems based on polymeric nanoparticles have been developed by pharmaceutical companies [20].

The objective of the present study was to address the knowledge on the use of nanosystems in aquaculture and possible toxic effects to fish. Here we prepared and characterized PLGA mucoadhesive nanocapsules containing BENZ (NPMAs) (5 mg/mL^-1^) and investigated the drug releases kinetics. Possible toxic effects in adults and embryos zebrafish exposed to the nanoformulations at distinct concentrations were also addressed. In addition, the interaction of the mucoadhesive system with external mucus of fish was determined and the anesthetic efficacy and the concentration of nanoparticles in tissue and water were measured. In summary this work presents the potential of mucoadhesive nanoparticles in effective drug release to fish.

## 2. Materials and methods

### 2.1. Preparation of chitosan-coated PLGA nanocapsules containing BENZ (NPMAs)

The NPMAs were prepared according to the interfacial polymer deposition method described by De Melo et al. [21] and Grillo et al. [22], with some modifications. 100 mg of PLGA (50:50) polymer, 40 mg of sorbitan monostearate, 200 mg of capric/caprylic triglyceride, 50 mg of BENZ and 0.1 mg of green fluorescent probe [1,2-distearoyl-sn-glycero-3-phosphoethanolamine-N-(7-nitro-2,1,3-benzoxadiazol-4-yl) ammonium salt] were dissolved in 30 mL of acetone. This phase was added under magnetic stirring to the aqueous phase, composed of 60 mg of polysorbate 80 and 30 mL of deionized water. The resulting emulsion was kept under stirring for 15 minutes and the organic solvent was removed under reduced pressure until the final volume reached 5 mL. The resulting suspension was poured into 5 mL of chitosan solution (5 mg.mL^-1^) under magnetic stirring during one hour for coating. The final suspensions were stored at room temperature in dark flasks [21, 22]. The final concentration of BENZ was 5 mg.mL^-1^ and final volume solution of 10 mL.

### 2.2. Encapsulation efficiency of BENZ in NPMAs

To determine the encapsulation efficiency of BENZ in the NPMAs, we employed the ultrafiltration/centrifugation method, which comprises centrifugation of suspensions using ultrafiltration devices with 10 kDa of MWCO (Amicon, Millipore). The ultrafiltrate was then quantified by high-performance liquid chromatography using the previously validated analytical curve equation [23]. The percentage of BENZ encapsulated was calculated by the difference between total and free drug concentrations.

### 2.3. Colloidal parameters of nanocapsules

The mean diameter and polydispersity index (PDI) were determined using the dynamic light scattering technique and zeta potential by microelectrophoresis. These measurements were performed at 25°C by diluting nanoparticle suspensions with purified water using a Zetasizer Nano ZS 90 particle analyzer (Malvern) with a fixed angle of 90 degrees. The size distribution was given by the polydispersity index. Results were expressed as the average of three determinations [24, 25].

### 2.4. Nanoparticle tracking analysis (NTA)

Nanocapsule concentration and size distribution were determined by employing the NTA technique. The suspensions were diluted 10,000 times and run in triplicate for each sample in a NanoSight LM10 instrument (Malvern) using a volumetric cell, 532 nm laser wavelength CMOS camera for image collection. The videos of Brownian motion were analyzed with the NanoSight 2.3 software. Each replicate consisted of five measurements of about 2,000 particles counted in each analysis [26, 27].

### 2.5. Nanocapsule morphology

The surface morphology of the nanocapsules was analyzed by Atomic force microscopy (AFM) and scanning electron microscopy (SEM). For AFM analysis, the suspensions were diluted 5,000 times and deposited on silicon plates previously prepared by removal of the silicon dioxide to facilitate interaction/adhesion of the sample. Analyses were performed with NanoSurf Easy Scan 2 microscope in non-contact mode [28]. For SEM analysis, the suspensions were diluted 2,000 times and the samples were mounted on aluminum stubs, sputtered with gold. Micrographs were obtained at an accelerating voltage of 5 KeV in a Quanta FEG 3D FEI scanning electron microscope [29].

### 2.6. Stability of nanosuspensions

The colloidal stability of the nanoparticles (NP) and NPMA suspensions was followed over time using measurements of particle size, polydispersity index, and zeta potential. The stability trials were continued for a period of 60 days, during which the formulations were stored at 25°C [25].

### 2.7. *In vitro* release assay

The release kinetics of BENZ from NPs and NPMAs was analyzed using a dialysis bag (cellulose membrane, 12 kDa MWCO) with donor (1 mL) and acceptor compartments (250 mL, distilled water). The systems were maintained under sink conditions and constant magnetic stirring. Samples were collected from the acceptor compartment for 2 hours. The experiment was carried out in triplicate and BENZ concentration was determined by HPLC. The drug release mechanism was also evaluated using the Baker-Lonsdale theoretical model, which is based on Fickian diffusion to elucidate drug release from spherical systems [24, 27].

### 2.8. *In vivo* assay

#### 2.8.1 Adult fish maintenance

For the *in vivo* assays, 90 male zebrafish, (≥ 4 months) from the same spawning were used, obtained from the Aquaculture Laboratory (Laqua) of the Veterinary School of the Federal University of Minas Gerais, Minas Gerais, Brazil. After transportation, the animals were acclimatized for 15 days and stored in aquaria with 2 L capacity, following the maintenance standards established by Westerfield [30]. The study was approved by the research ethics committee of UFMG (protocol CEUA-UFMG 336/2017). All experiments were performed in accordance with relevant guidelines and regulations.

#### 2.8.2 Evaluation of nanocapsules fluorescence in zebrafish adults

Adult zebrafish (n = 10) was individually exposed immersed for 5 minutes in a becker of 500 mL containing NPMA in concentration 0.050 or 0.025 mg.mL-1, in accordance with protocols described by Charlie-Silva et al. [31]. After the time of immersion, the zebrafish were transferred to a becker with 500 mL of clean water to be measured the interaction of NPMA and fish surface. Immediately after anesthesia were analyzed in vivo fluorescence images produced by nanocapsules that were absorbed by zebrafish tissues using a Bruker Xtreme In-Vivo Imaging System (Bruker, Billerica, MA, USA). Fish liver, gill and intestine images confocal intravital microscopy was performed as previously described by Marques et al. [32]. The counts were performed on the images captured in the confocal microscope at MS FXPRO (Bruker).

#### 2.8.3 Evaluation of anesthesia: Induction and recovery

Adults zebrafish were individually anesthetized by immersion in solution with mucoadhesive NPMA. This solution was prepared with 0.125, 0.050 and 0.025 mg.mL^-1^ of NPMA in a final volume of 500 mL water. Anesthetic induction and recovery time was registered, using a stopwatch. During the experiments, the loss of reflexes to external stimuli and absence of opercular movement were used to characterize the state of anesthesia. Then each zebrafish was placed in a beaker with 1 L of clean water for recovery. Fish were considered recovered when they had normal balance and reaction to external stimuli [33]. At the end of the evaluations, fish from each treatment were transferred to 3 L aquaria (Science Labor Glassware) with recirculating water and observed clinical sings and sampled for histological analysis (n = 10) at 24 and 96 hours post- anesthesia.

#### 2.8.4 Histopathological evaluation

The fish adults were exposed to BENZ, NP and NPMA for 5minutes to concentrations of 25 and 50 mg.L^-1^ and after 24 and 96 hours, zebrafish were necropsied to collect liver, kidney, spleen, and gills for histopathological analysis. Sections with 6 μm thickness were mounted on slides and stained with hematoxylin-eosin (HE) for observation of general cell structures [34].

#### 2.8.5 Embryo acute toxicity

This test was based on the OECD guideline on Fish Embryo Toxicity (FET) Test [35]. Zebrafish eggs were produced by the Central Biotery of the Federal University of São Carlos according to “The zebrafish book” [30]. The embryos were collected 10 min after natural mating, rinsed with embryo media, E3 [36], and selected under a stereomicroscope for viability for the assay i.e., fertilized and not showing abnormal cleavage. Eggs with an opaque appearance were considered dead and not included in the experiment. The eggs were distributed in a 96-well plate containing 200 μL of the E3, with several dilutions of NP, PLGBMA NPMA, and BENZ: 3.9, 15.6 and 62.5 μg.mL^-1^. Twenty replicates of each concentration were performed, i.e., one egg per well as one replicate. Negative controls were also conducted in each plate and a separate plate. The eggs were incubated at 28°C for 96 h. Every 24 h they were checked under the stereomicroscope and the main endpoints recorded were survival, hatching, tail detachment, and presence of edema [35].

### 2.9. Statistical analysis

The normality of the data was measured using the Shapiro-Wilk test. After analysis of normality, the experimental data were submitted to analysis of variance in completely randomized design subdivided in time (split-plot design), using the statistical software package R. The survival curves were analyzed by Kaplan-Meier method using the log-rank (Mantel-Cox) statistical test to compare the groups. The means were compared by the Bonferroni test at 5% significance level and graphs were plotted with the GraphPad Prism 7 software for Windows.

## 3. Results and discussion

### 3.1. Colloidal parameters

Mucoadhesive polymeric nanocapsules were prepared with the model drug BENZ. In this study, NPs and NPMAs containing BENZ (5 mg.mL^-1^) were prepared and characterized in terms of colloidal parameters and are represented in **Table 1**. The aim of the coating was to provide mucoadhesive properties to the nanocapsules. After preparation, the suspensions were characterized according to mean diameter, polydispersity index, zeta potential, and nanoparticle concentration. The nanoparticles were obtained with a mean diameter between 200 and 300 nm and a polydispersity index below 0.2. The mean diameters and polydispersities of NPs and NPMAs were compatible with those commonly found for polymeric nanosystems studied previously [14, 21, 37]. There was an increase in NPMA diameter, providing evidence of coating formation with no aggregation of nanoparticles. The concentration of chitosan used in the coating solution (5 mg/mL^-1^) provided nanoparticles with a diameter very close to those reported in the literature, with narrow size distribution and thick coating, demonstrating good interaction between the coating layer and the nanocapsule polymer wall [22]. The polydispersity index values were below 0.2 for both nanocapsules, indicating a homogeneous particle diameter distribution.

The NTA technique was used to measure nanoparticle concentration (**Table 1**). For NPs, the concentration was 1.19 ± 0.44 × 10^17^ particles/mL^-1^, with a mean diameter of 190 ± 50 nm. For NPMAs, the concentration was 1.11 ± 0.29 × 10^17^ particles/mL^-1^, with an average diameter of 195 ± 59 nm. Both DLS and NTA techniques provided similar mean diameter values and the same particle concentration range, indicating homogeneity of the system, which is desirable in nanoparticle formulations since it reduces interferences with drug-release by increasing stability.

**Table 1:** Initial values of mean diameter (nm), polydispersity index (PDI), zeta potential and encapsulation efficiency of suspensions: NP (content benzocaine), NP-control, NPMA (content benzocaine) and NPMA-control (n = 5).

Particle	Content BENZ	Mean diameter (nm); (PDI)	Zeta potential (mV)	BENZ encapsulation (%)	Concentration (particles.mL^-1^)
NP	BENZ	249.2 (0.144)	- 25.5	84.8	1.19 x 10^17^
NP-control	No	200.1 (0.157)	-19.5	-	1.45 x 10^17^
NPMA	BENZ	297.5 (0.185)	+44.3	72.1	1.16 x 10^17^
NPMA-control	No	263.7 (0.191)	+46.7	-	1.11 x 10^17^

The zeta potential values obtained for NPs, NPMAs and respective controls had values between -19.2 and + 46.7 mV, showing that the addition of the chitosan promoted changes of values due to the presence of positively charged amino groups, indicating successful coating of the nanoparticle surface. Similar results were found for chitosan-coated polymer and zein nanoparticles [22, 38].

The encapsulation efficiency of BENZ was 84.8% for NPs and 72.1% for NPMAs. In previous studies, characterization of different nanocapsules containing BENZ (4 mg.mL^-1^) formulations has revealed an encapsulation efficiency between 70 and 73%, average size of 130–160 nm and negative zeta potential [39]. This difference is attributed to the process of the nanoparticles. Probably BENZ molecules that would be on the surface of the nanocapsules were displaced by chitosan, increasing the amount of free drug in the system and resulting in slightly lower encapsulation efficiency. Similar results were also found for chitosan-coated PCL nanocapsules containing the herbicide atrazine [22]. Nevertheless, the amount of encapsulated BENZ was considered very satisfactory.

### 3.2. Morphological characterization of NPMA nanocapsules

**Fig 1:**
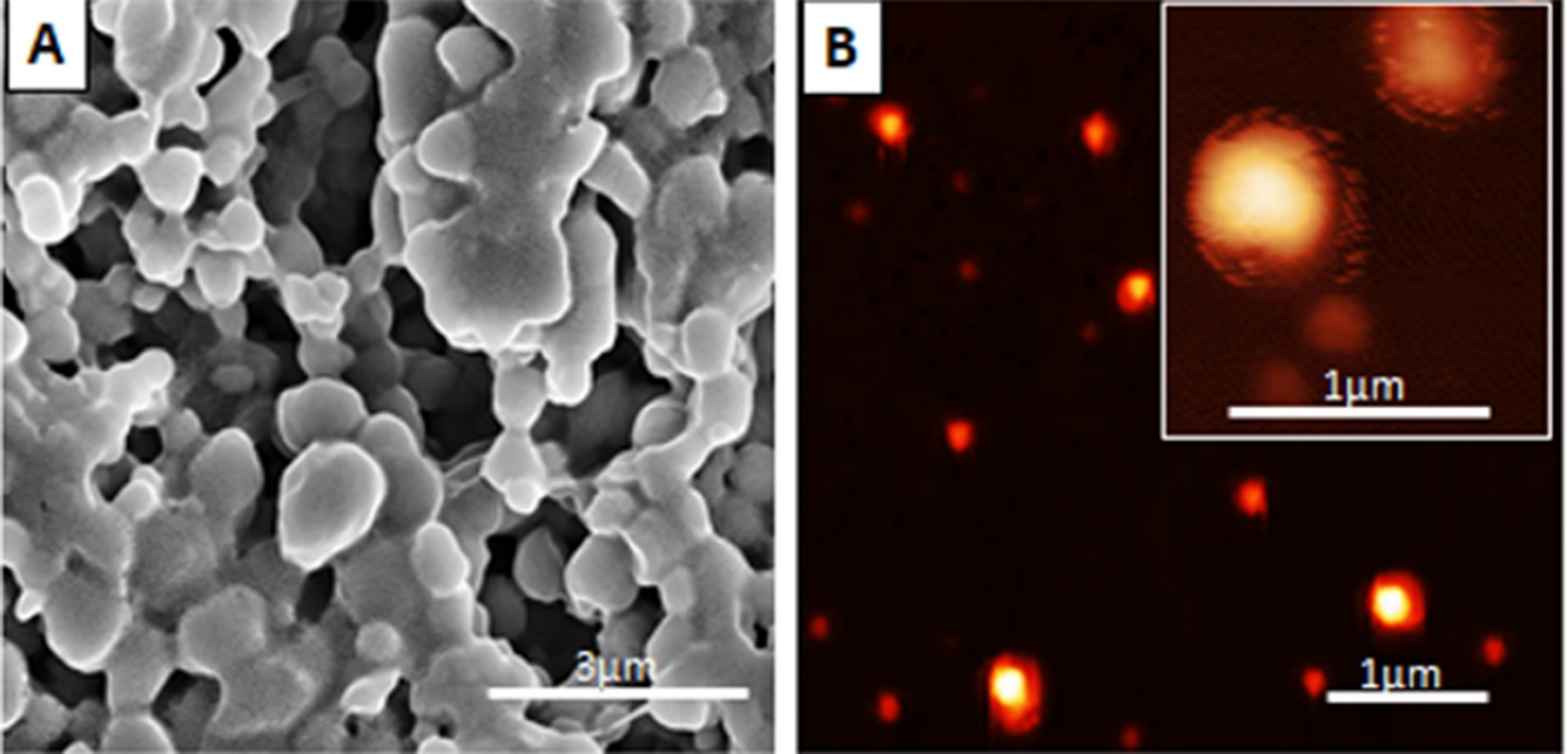
Micrographs of NPMAs. **(A)** For scanning electron microscopy (SEM), NPMAs were diluted 1:5000 and mounted on plastic coverslips (Thermanox), metalized with gold in an evaporator (Bal-Tec, model MD20) and analyzed by SEM with secondary electrons, backscattered electron imaging and **(B)** Atomic force microscopy (AFM) image using non-contact mode, sample diluted 1:5000 and deposited in silicon plates.

We studied the morphology of the nanocapsules by atomic force microscopy (AFM) and scanning electron microscopy (SEM). According to the AFM and SEM images (**Fig 1**), the nanoparticles were spherical with diameters in the range of 180–300 nm, with no agglomerates. Particle sizes were compatible with the mean diameter values obtained by the DLS and NTA techniques.

### 3.3. Stability of nanosuspensions

**Fig 2:**
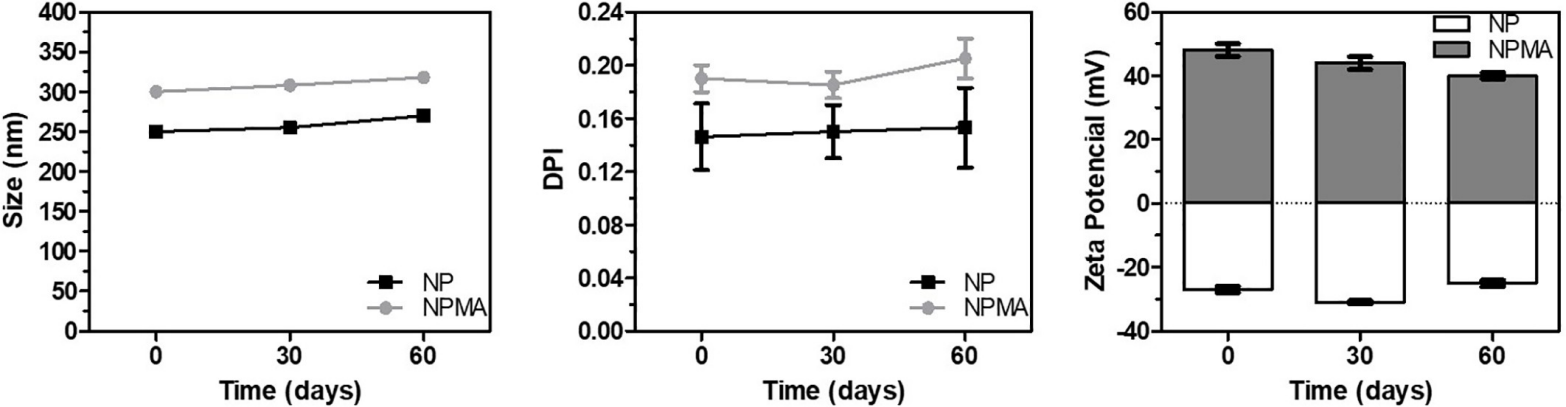
Colloidal stability of NPs and NPMAs. Colloidal parameters as size, PDI and zeta potential were evaluated as a function of time using the DLS technique (n = 3). All parameters were considered stable, indicating the suitability of the nanoformulations.

In **Fig 2** the sizes of particles of the two nanosuspensions analyzed remained almost constant for a period of 60 days, with no evidence of aggregates. During the trial period, none of the formulations exhibited PI values over 0.2, indicating satisfactory colloidal stability parameters. Zeta potential values also remained constant, indicating electrostatic stabilization of nanoparticles. Several works have reported suitable colloidal stability of polymeric nanocapsules containing local anesthetics [14, 21, 24, 40]. Based on the literature, we can assume that the nanocapsules developed here presented colloidal stability as a function of time and was prepared for in vitro release and in vivo experiments.

### 3.4. *In vitro* release kinetics

**Fig 3:**
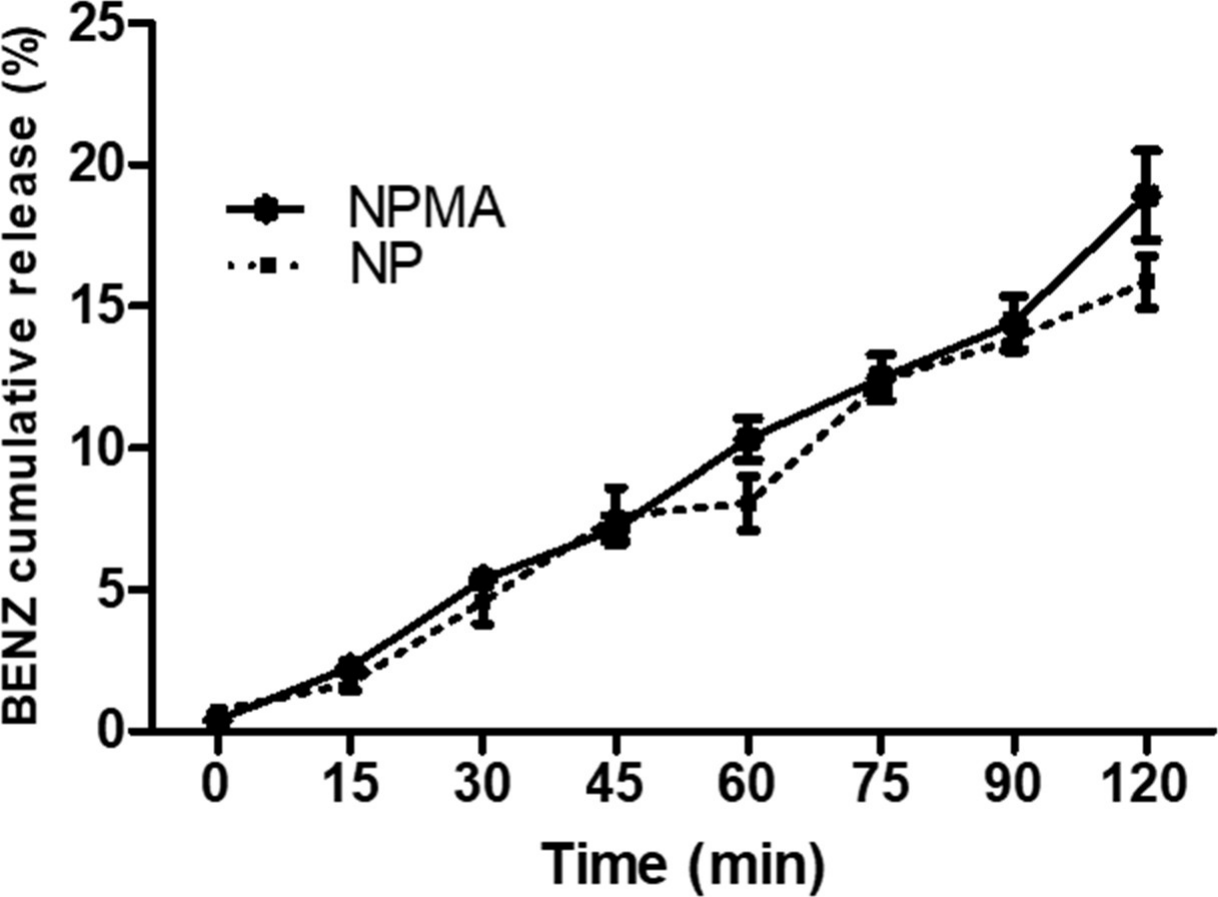
Release kinetics of BENZ in NPs and NPMAs. The systems were maintained under magnetic stirring. Samples were collected during 2 hours and BENZ concentration was determined by HPLC. The experiments were conducted in triplicate at room temperature. The percentage of BENZ released was less than 20% for both nanocapsules, showing sustained drug release. No statistical difference was observed for the BENZ release curves (*p>0.05).

The BENZ release was investigated using two compartments separated by a cellulose membrane (12kDa MWCO). The kinetics assay provided information about the interaction between drugs and nanocapsules as well as the mechanism of drug release. Aliquots were collected from the acceptor compartment for 2 hours and the results were expressed as percentage of BENZ released (**Fig 3**).

The drug release profile is vitally important in terms of effective therapy, providing information on release mechanisms and interaction between pharmaceuticals and nanocarriers [25]. The **Fig 3** shows the release profiles of BENZ encapsulated in NPs and NPMAs, at room temperature (25°C) as a function of time. Only free drug can pass unhindered through cellulose membrane. None of the formulations were able to release more than 20% of the anesthetic in aqueous media during 120 minutes. These findings indicate that the encapsulation of BENZ in polymeric nanocapsules was suitable to modify the drug release profile, allowing only small amounts of the drug to be released into the water. This can be an advantage compared with the free drug dissolved in the immersion bath for fish anesthesia. The nanoparticulate mucoadhesive system (NPMA), when dispersed in water, has strong interaction with the outer mucus layer of the fish, promoting a more targeted release of BENZ and more effective anesthesia, while the free anesthetic solution is susceptible to hydrolysis even before absorption by the fish [10, 39]. Comparing the two nanocapsules (NPs and NPMAs), no statistical difference was observed between the curves (*p>0.05). Regarding the encapsulation efficiency values, we observed a tendency for faster release of BENZ from NPMAs, which presented 72.1% drug encapsulation efficiency (84.8% for NPs), suggesting a relationship with the amount of active compound encapsulated and release profile. Similar results can be found in the literature for nanoparticles containing local anesthetics and herbicides [22, 24, 40].

Mathematical modeling was employed to elucidate the drug release from the nanoparticle systems. The Baker-Lonsdale model has been used previously by our group to evaluate the results of release curves involving nanoparticles [24, 25, 27]. Linear regression showed correlation coefficient (*r*) values of 0.978 and 0.988, and release constant (*k*) values of 4.24822 x 10^−5^ (± 2.83661 x 10^−6^) min^-1^ and 6.65378x 10^−5^ (± 4.27674 x 10^−6^) min^-1^, for NPs and NPMAs, respectively. Through mathematical fitting, it was possible to compare the two release curves, and the constant values (*k*) demonstrated that BENZ is released faster from NPMAs than NPs due to the presence of chitosan coating, which probably translocates BENZ molecules from the surface. The values of *r* indicated the main mechanism involved in the release of the drug was Fickian diffusion, as also found previously for other polymeric nanoparticles containing articaine [24, 25] and solid lipid nanoparticles containing prostaglandin J_2_ [27]_._

### 3.5. Anesthetic induction and recovery in adult zebrafish

**Fig 4:**
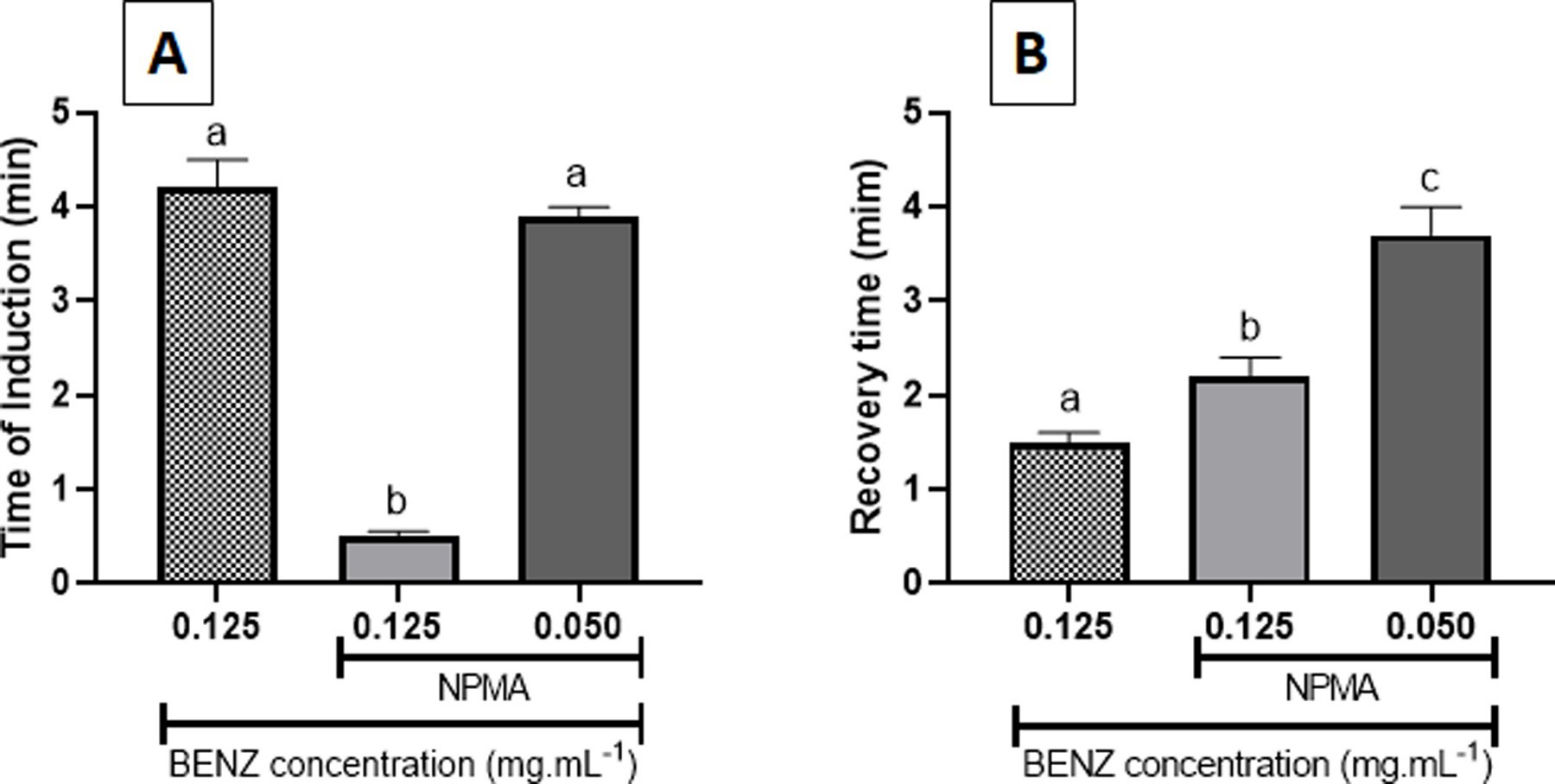
Evaluation of time for anesthesia induction and recovery of zebrafish adults exposed to BENZ and NPMA. **(A)** Anesthetic induction time **(B)** Recovery time of zebrafish adults.

The use of nanocapsules containing BENZ at different concentrations influenced the anesthetic delivery in zebrafish adults, when tested along time. Zebrafish anesthetized with NPMA at concentration of 0.125 mg.mL-1 showed significant (p<0,05) decrease in the time of induction and significant (p<0,05) increase the recovery time when compared to fish anesthetized with BENZ at the same concentration (0.125 mg.mL-1) (**Fig 4**). Zebrafish anesthetized with NPMA at the concentration of 0.050 mg.mL-1 showed mean values of 3.38 min. for induction, and no significant difference (p>0.05) was observed when compared to BENZ (0.125 mg.mL-1) (**Fig 4**). However, both NPMA concentrations (0.125 and 0.050 mg.mL-1) anesthetic induction resulted in a significant increase in the animals' recovery time compared to fish anesthetized with conventional benzocaine (BENZ) (**Fig 4**). The lowest concentration (0.025 mg.mL-1) resulted in the longest time for the animal to be induced (13.17min.) to the anesthetic plane. This long period of anesthetic induction is not desired in zebrafish anesthesia procedures, so these data have been discarded (data not shown). The concentration of 0.050 mg.mL-1 resulted in increase in the induction time for the animal to be induced (13.17min.) to the anesthetic plane and to return to stability, while at the highest concentration (0.125 mg.mL-1), the time required for induction and recovery was lower (**Fig 4**). From these results, it is possible to observe a relationship between the BENZ encapsulation, being able to modify the release profile of the anesthetic and the mucoadhesive property of the NPMA, enabling the efficient delivery of the anesthetic in the fish tissues, promoting anesthesia. Zebrafish exposed to mucoadhesive nanocapsules without BENZ showed no anesthetic effect after 24 and 48 hours exposure.

### 3.6. The nanoparticles were absorbed in adult zebrafish tissues

**Fig 5:**
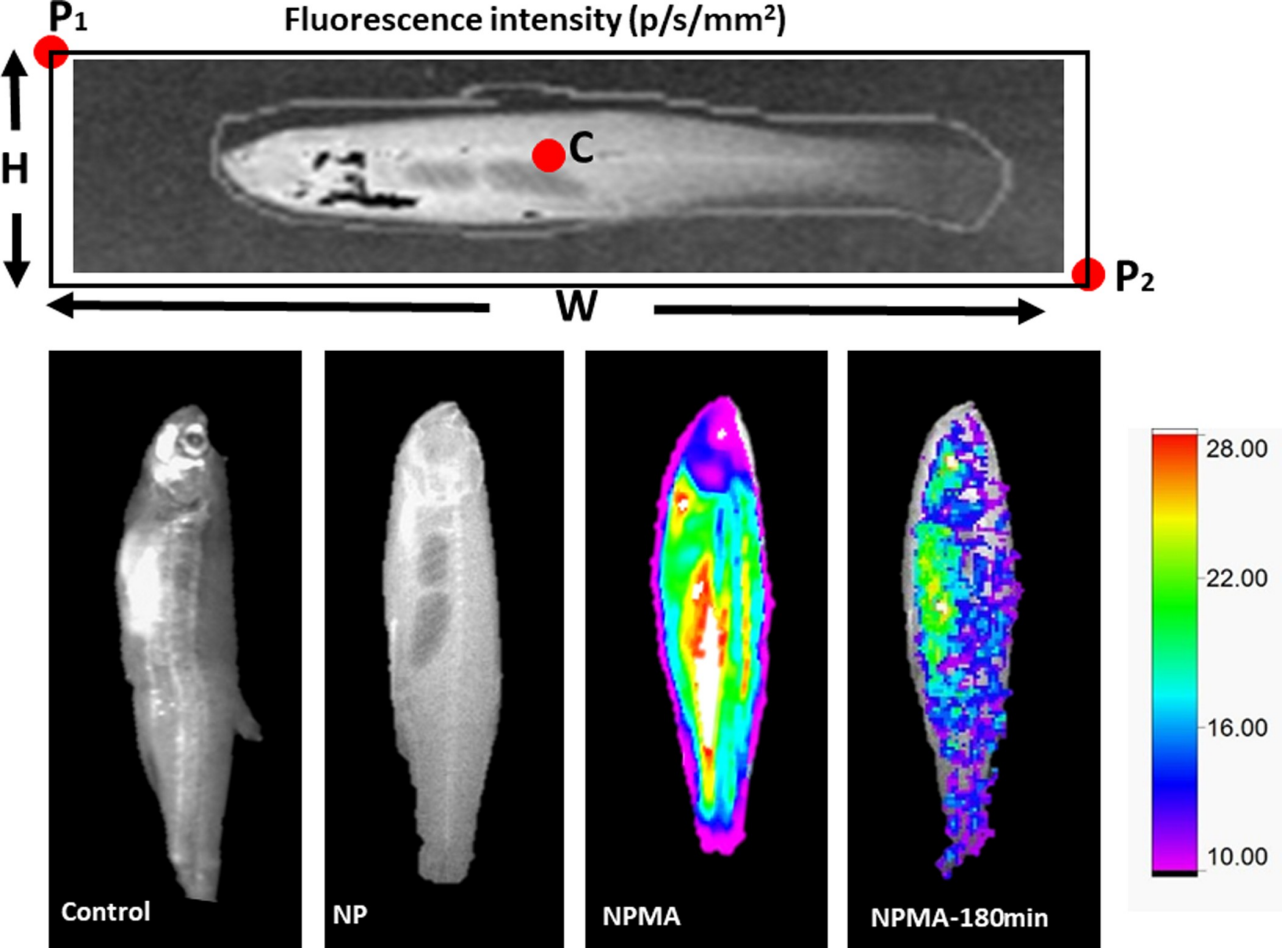
Interaction between mucoadhesive nanocapsules and zebrafish. *In vivo* fluorescence images of zebrafish exposes to NPMAs.

**Fig 6:**
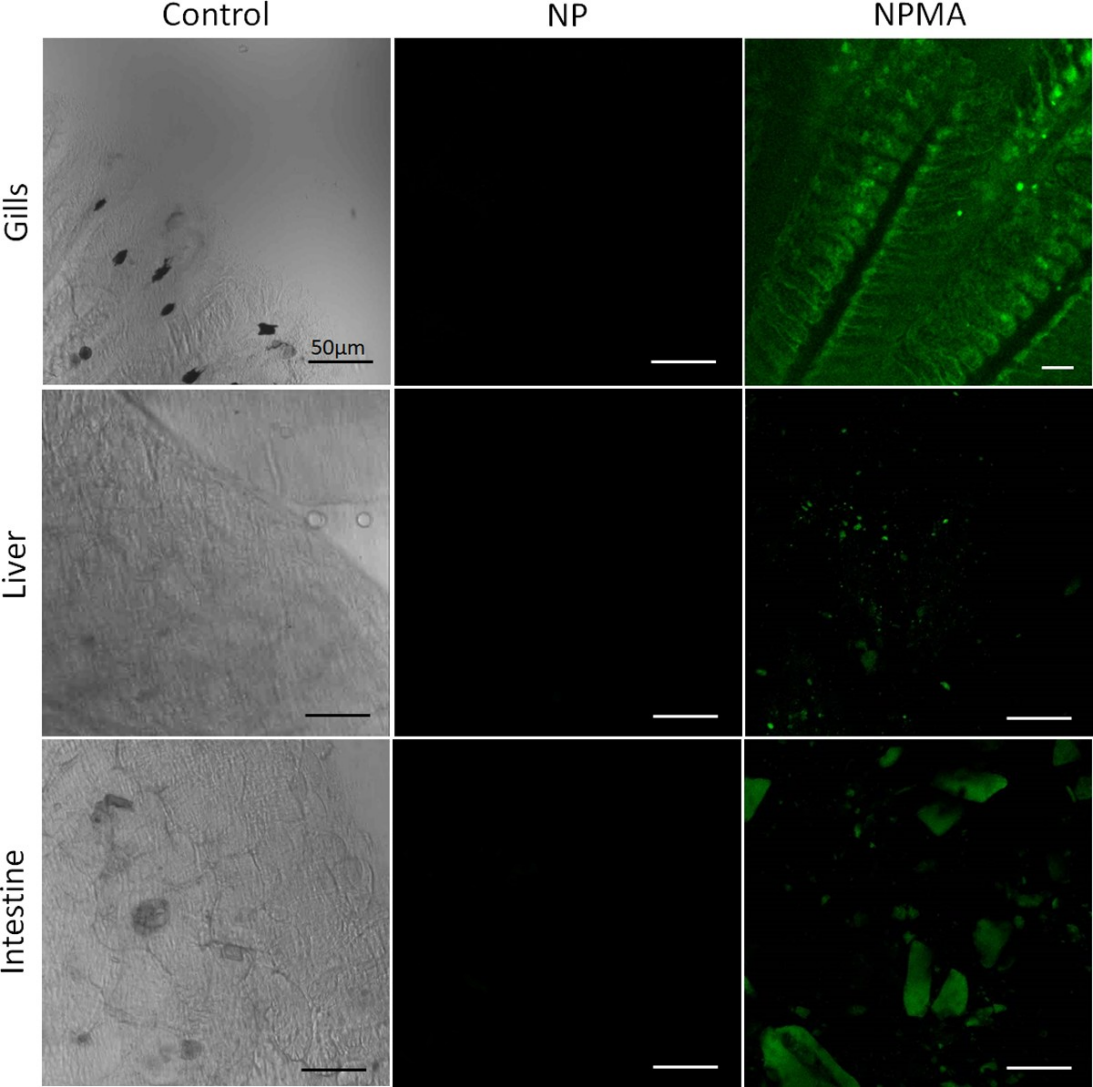
Non-invasive confocal imaging of zebrafish structures after exposure to NP, NPMA or BENZ. Fish gills, liver and intestine images showed fluorescence only under NPMA treatment.

We measured the intensity of NPMAs nanocapsules fluorescence in the tissues immediately after anesthesia exposure and 180 minutes later (**Fig 5**), and we found 138 and 57 p / s / mm² for 0.025 mg/mL^-1^, respectively. **Fig 6** shows fluorescence obtained as a function of nanoparticle concentrations. These results demonstrated, through the tracking of the fluorescent probe, that the mucoadhesive nanocapsules containing benzocaine (NPMAs) were able to establish an interaction with the mucous surface of the fish, permeating the tissues of the skin.

In addition, for evaluation of fluorescence in the tissue, fragments of gill, liver, and intestine were collected and processed. The organs were removed, and sections were mounted on slide glass for observation of the general cellular structures by confocal microscopy. We observed the predominance of fluorescence in the gills followed by liver and intestine (**Fig 6**). From these findings, we can assume that NPMA suspension in contact with the fish allowed the absorption of these nanoparticles by the tissues. One of the possible mechanism involved in nanoparticle internalization is endocytosis [41]. Similarly, we can also infer that the absorption of NPMAs through the gills (**Fig 6**), permitting the nanosystems to exhibit an improved pharmacokinetic profile, and, consequently, superior pharmacological activity.

### 3.7. NPMA was not toxic to adult zebrafish tissues

**Fig 7:**
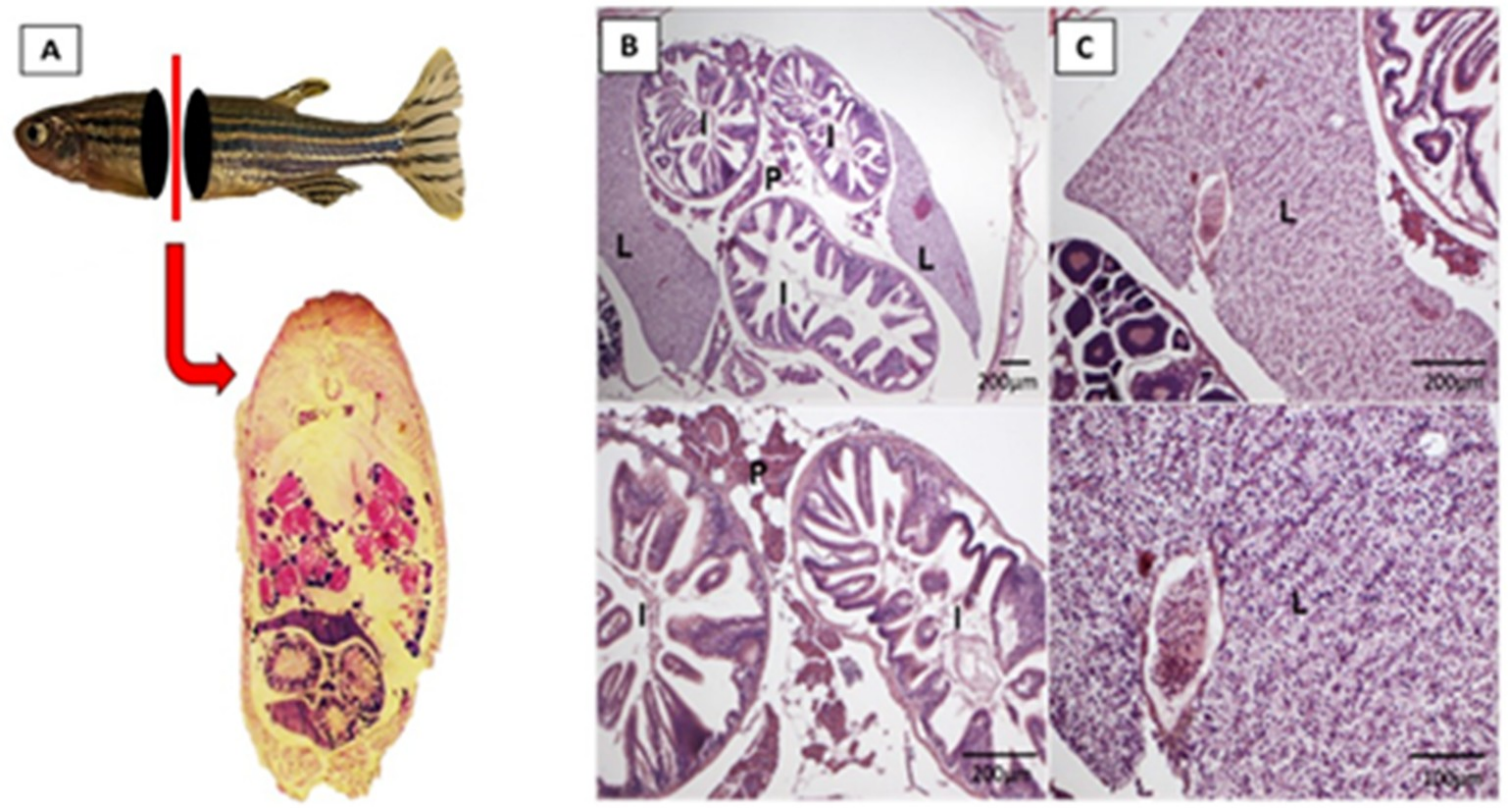
Histopathological analysis of fish exposed to NPMAs: Adult zebrafish exposure to NPMAs at concentrations of 25 and 50 mg/L^-1^. (A), (B) and (C) show fish sections without relevant pathological alterations. (L) liver; (P) pancreas and (I) intestine (H&E staining).

Adult fish exposed to BENZ, NP and NPMA were prepared for histological analysis. Tissues of adult organs of fish were prepared for sections (6 μm) and H&E-stained. The slides were examined by light microscopy and no histopathological damage was found (**Fig 7**). Fish anesthesia with BENZ, NP and NPMA at 25 and 50mg.L^-1^ did not lead to histological injuries (**Fig 7**). Zebrafish are more sensitive to benzocaine with age. In the literature zebrafish embryos exposed to benzocaine at 200mg.L^-1^ for 24h and 5 days post fertilization (dpf) larvae for 2h and had a survival rate of above 80%, while adult fish of 9-11month-old exposed for 15min had a survival rate of around 40%, demonstrating an increased sensitivity to anaesthetics along ageing [42]. Euthanasia in different fish species using benzocaine is considered effective at 100mg/L [43]. Young fish of 42-47days did not survive to 50mg.L^-1^ benzocaine exposure longer than 56min [44] and more than 50% of zebrafish adults anesthetized with 35mg.L^-1^ benzocaine died in a continuous exposure periods longer than 6h [45]. Although the period of incubation with BENZ, NP and NPMA was short, 5minutes, it was the half concentration of euthanasia and did not affect the analyzed organs. In view of this, the nanosystems here developed presented low tissue toxicity, showing potential safety of these nanocarriers for drug delivery.

### 3.8. Toxicity assay for NP and NPMA in zebrafish embryos

**Fig 8:**
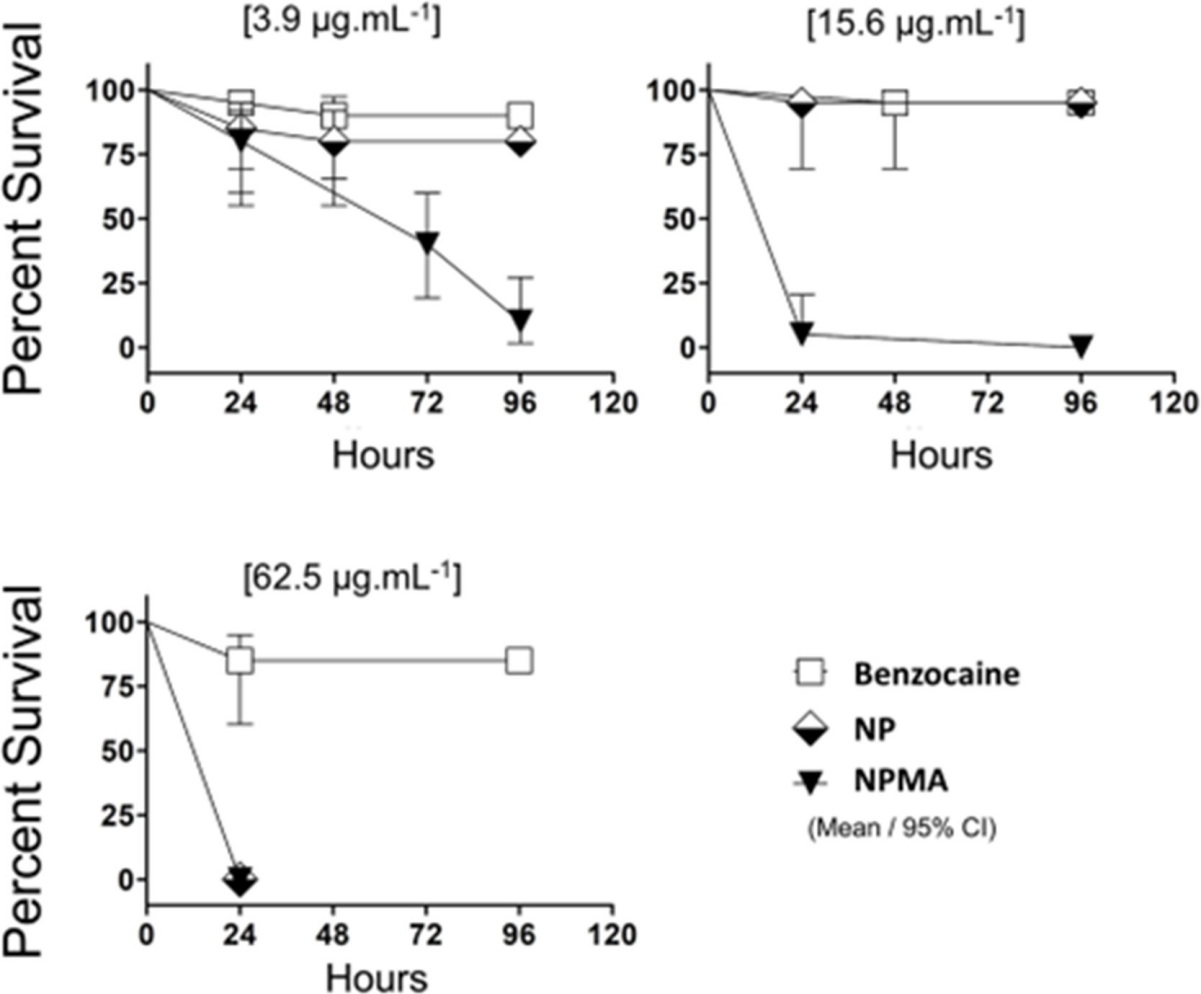
Survival percentage of embryos exposed to free BENZ and encapsulated in nanocapsules. Survival rate curves of embryos exposed to BENZ, NP and NPMA at concentrations of 3.9, 15.6 and 62.5 μg/L^-1^. The Kaplan Meier curve was constructed and performed the log-rank (Mantel-Cox) statistical test to compare the groups.

In order to analyze in vivo toxicity of encapsulated BENZ, the Fish Embryo (FET) test was performed in zebrafish embryos exposed to three different BENZ, NP and NPMA concentrations for 96h. BENZ in solution did not affect the survival rate at any concentration tested during the entire experiment (**Fig 8**). NP was significantly toxic to embryos in the highest concentration tested, 62.5μg.mL^-1^ at 24h (**Fig 8**). However, embryos exposed to NPMA, even to the lowest concentration tested, 3.9μg/mL^-1^, died and demonstrated an decreasing survival rate through time (minimum survival of 15% after 96 hours) (**Fig 8**). All embryos from the control groups showed normal development at 96 hpf (data not shown).

**Fig 9:**
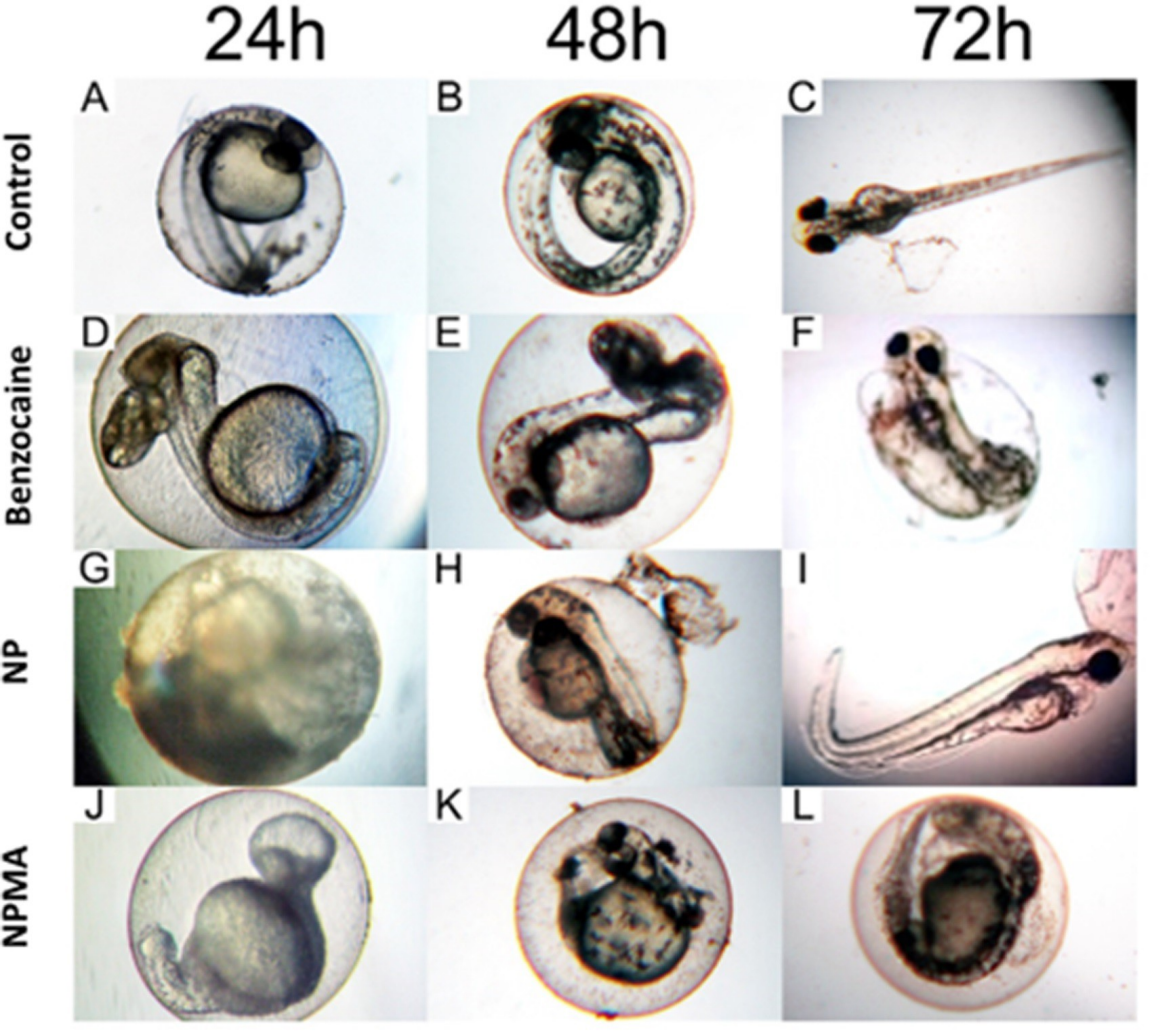
Sublethal effects of zebrafish embryos exposed to NP, NPMA, and BENZ. (A-C) control embryos presenting wildtype morphology. (D-F) Embryos exposed to BENZ revealed tail malformation (D and E) and pericardial edema (F). (G-I) Embryo contact with NP developed curved tail. (G and H) Nanoparticles formed aggregates deposition on the chorion surface. (J-L) NPMA exposure led to general malformation of embryos at 3.9 μg/L^-1^.

Some sublethal effects were observed during the exposure to NP, NPMA, and BENZ (**Fig 9**). Although the anesthetic solution did not generate significant mortality, at the highest concentrations (15.6 and 62,5μg.mL^-1^) over 75% of the larvae had tail malformation exposed to BENZ at 24 and 48h (**Fig 9D and 9E**), and at 72h developed pericardial edema (**Fig 9F**). The predominant effects of NP was a curved tail at higher concentrations (**Fig 9G–9I**). However in **Fig 9G** is almost not possible to observe the phenotype of the embryo because of the accumulation and adhesion of the nanoparticles on the surface of the chorion. Those aggregates on the chorion were also found in NPMA treatments (data not shown). The formation of these aggregates depended on particles sizes, shapes and coating materials that might interact differently with biological molecules such as membrane proteins and DNA [46]. Future works could reveal if the nanoparticles interact with extracellular protein structures, such as chorion proteins. Concentrations of NP and BENZ below 15.6 μg.mL^-1^ did not affect embryo development. More interestingly, BENZ concentrations that were safe for the embryos were highly toxic when combined with the mucoadhesive nanocapsules. The embryos that survived the NPMA exposure at the lowest concentration 3.9μg.mL^-1^ showed severe malformations from 24-72h (**Fig 9J–9L**). Demonstrating that the toxic effects of benzocaine were magnified by the mucoadhesive nanoencapsulation.

In the literature, zebrafish embryos were exposed to benzocaine at 200mg.L^-1^ for 24h and 5 days post fertilization (dpf) larvae for 2h and had a survival rate of above 80% [47]. This work demonstrated that encapsulated benzocaine (NPMA) exposure to embryos was 50x more toxic than the literature (comparing 200mg.L^-1^ with 3.9μg.mL^-1^). Possibly the nanoparticles facilitated the entry of BENZ through the chorion, so the association of nanoparticles and BENZ increased the anesthetic exposure, which increased drug delivery and toxicity to embryonic development.

## 4. Conclusion

The chitosan-coated mucoadhesive nanocapsules (NPMAs) containing BENZ demonstrated enhanced anesthetic performance in fishes. An important feature is the slower release profile of the drug, allowing the use of low concentrations of the BENZ with good anesthetic induction and recovery time. In contrast, free BENZ was immediately subject to hydrolysis and absorption, leading to greater problems related to contamination and intoxication. The toxic effects in embryos exposed to the nanoformulations occurred because of the facilitated absorption of BENZ in nanoparticles impairing embryonic development, compared with free drug. Analysis of the NPMAs showed the release is directed to fish tissues, particularly the surface, so there are no losses of the drug, allowing administration of lower doses. The exposure of the tissues to NPMAs did not cause visible damage in the histopathological analysis suggesting low toxicity of nanoparticles in adult zebrafishes. Regarding this, we demonstrated the nanoencapsulation of benzocaine increases its absorption, as well as protects from degradation, enhancing its effects. Therefore, the formulations presented in this study prospect the use of BENZ nanoformulations in aquaculture, allowing reduction of dosage and promoting more effective anesthesia of fish.

## Supporting information

S1 File (PDF)
