# Supplementary material for: Potential of mucoadhesive nanocapsules in drug release and toxicology in zebrafish
Source: PLoS One. 2020 Sep 24;15(9):e0238823. doi: 10.1371/journal.pone.0238823 (PMC7514080; doi:10.1371/journal.pone.0238823)
Supplement: S1 File — (PDF) [file pone.0238823.s001.pdf]

Supplementary information

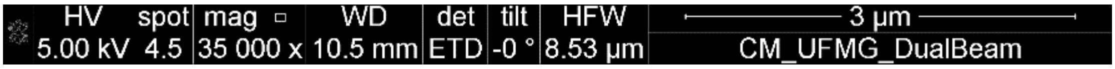

Micrographs of NPMAs. Data For scanning electron microscopy (SEM),

Table. Initial values of mean diameter (nm), polydispersity index (PDI), zeta potential and encapsulation efficiency of nanosuspensions: NP (content benzocaine), NP-control, NPMA (content benzocaine) and NPMA-control (n=5).

sParticles	Mean diameter (nm); (PDI)	Zeta potential (mV)	BENZ encapsulation (%)	Concentration (particles.mL^{-1})
NP	249 ± 2 (0.14 ± 0.02)	- 25.5 ± 1.4	84.8 ± 1.2	1.1 x 10^{17} ± 4 x 10^{16}
NP-control	200 ± 1 (0.15 ± 0.03)	-19.5 ± 0.8	-	1.4 x 10^{17} ± 1 x 10^{16}
NPMA	297 ± 5 (0.18 ± 0.01)	+44.3 ± 0.5	72.1 ± 0.9	1.2 x 10^{17}± 2 x 10^{16}
NPMA-control	263 ± 2 (0.19 ± 0.01)	+46.7 ± 1.1	-	1.3 x 10^{17}± 1 x 10^{16}

Interaction between mucoadhesive nanocapsules and zebrafish. *In vivo* fluorescence images of zebrafish exposes to NPMAs.

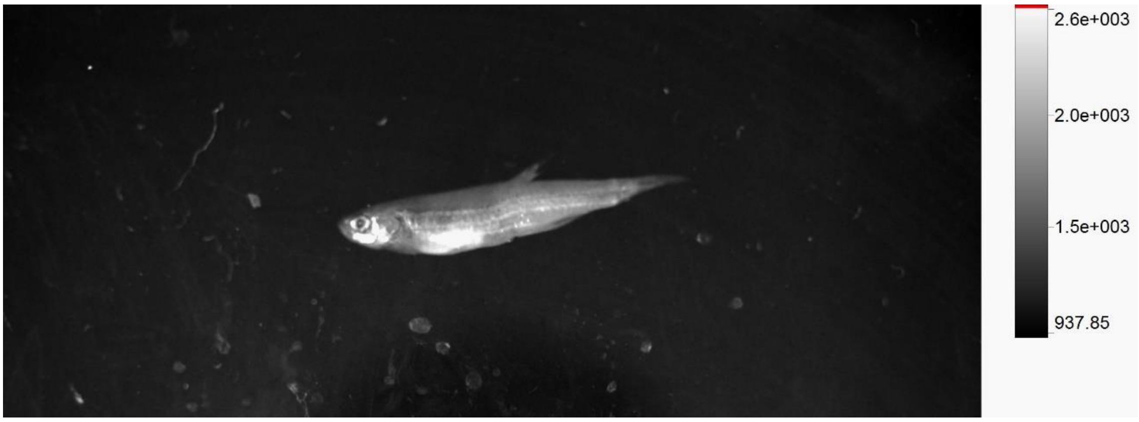

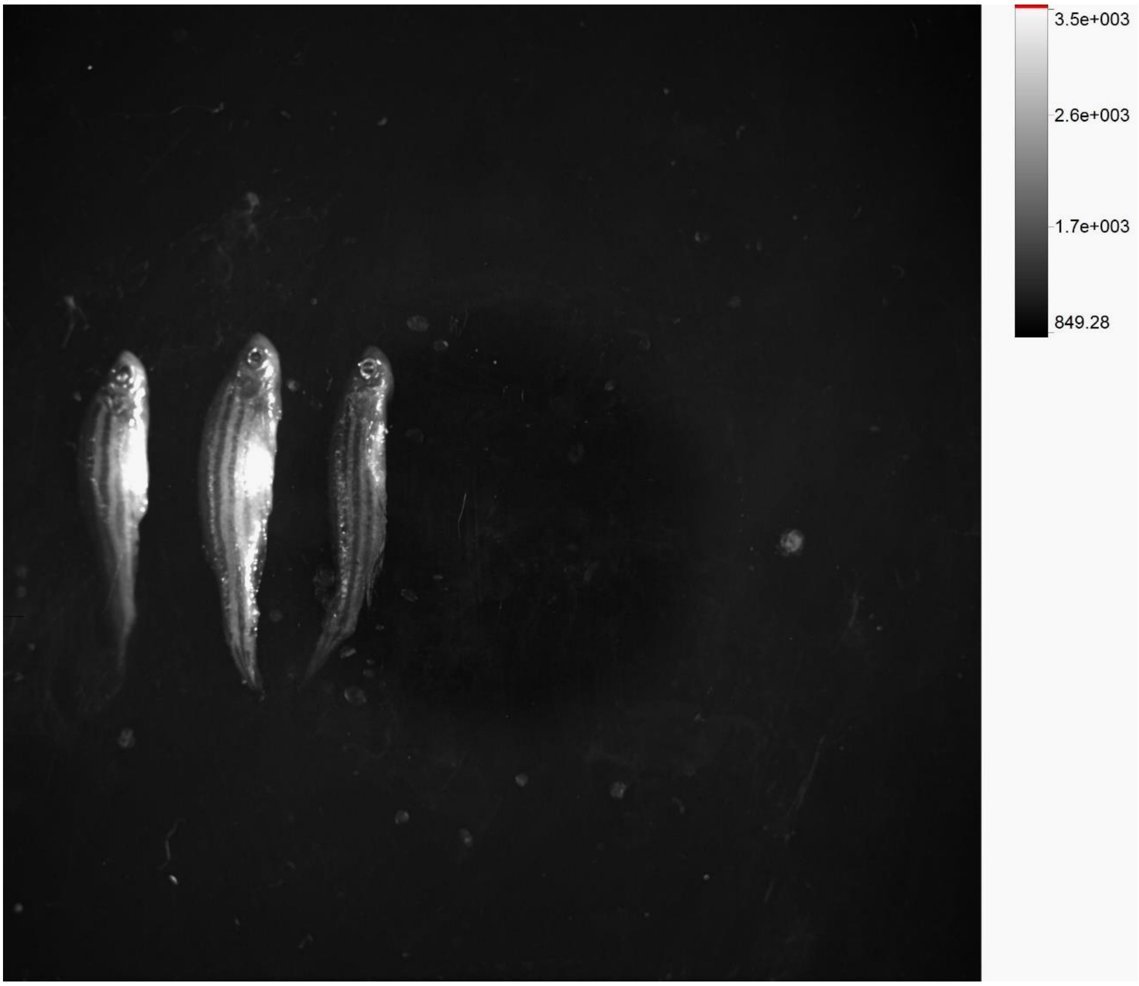
3.5e+003
2.6e+003
1.7e+003
849.28

Supplementary information

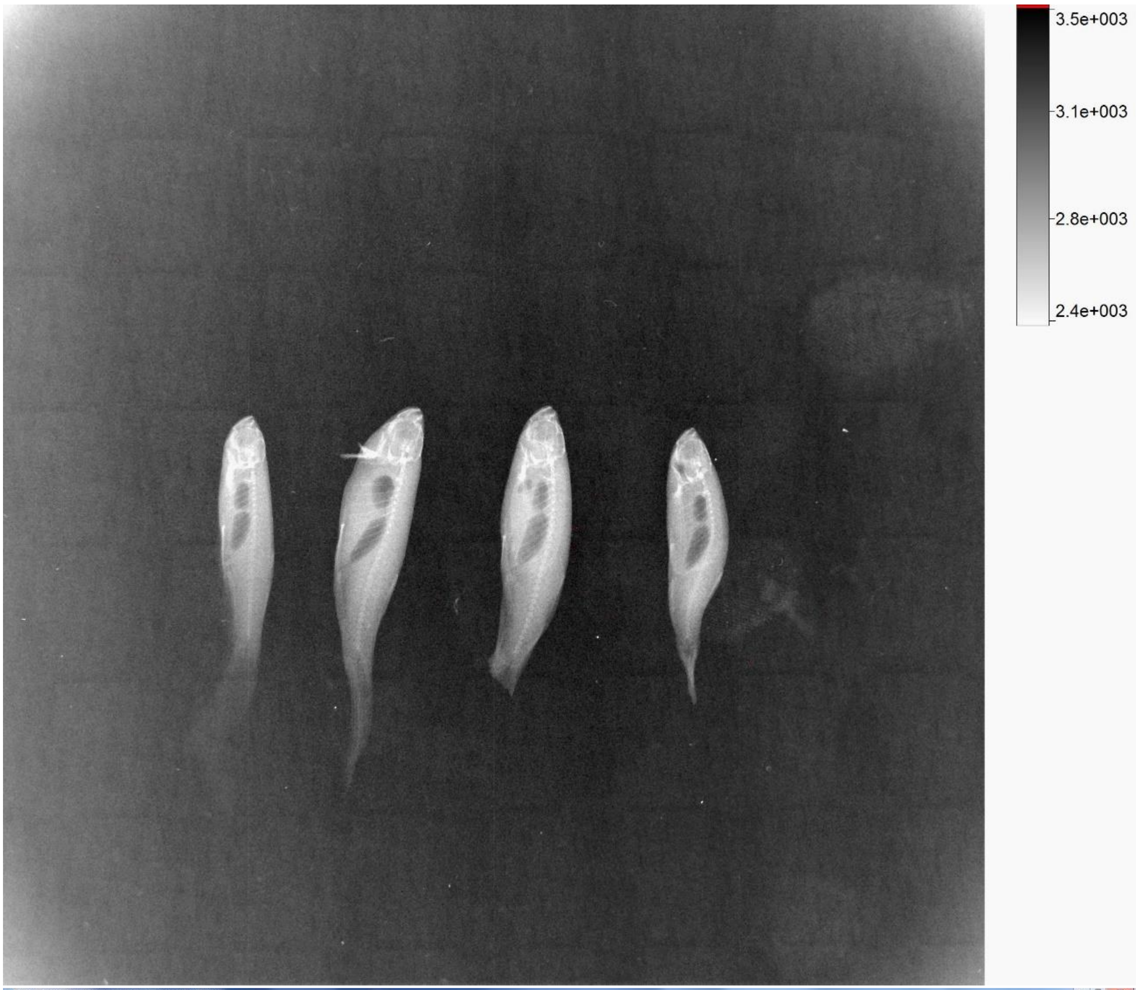

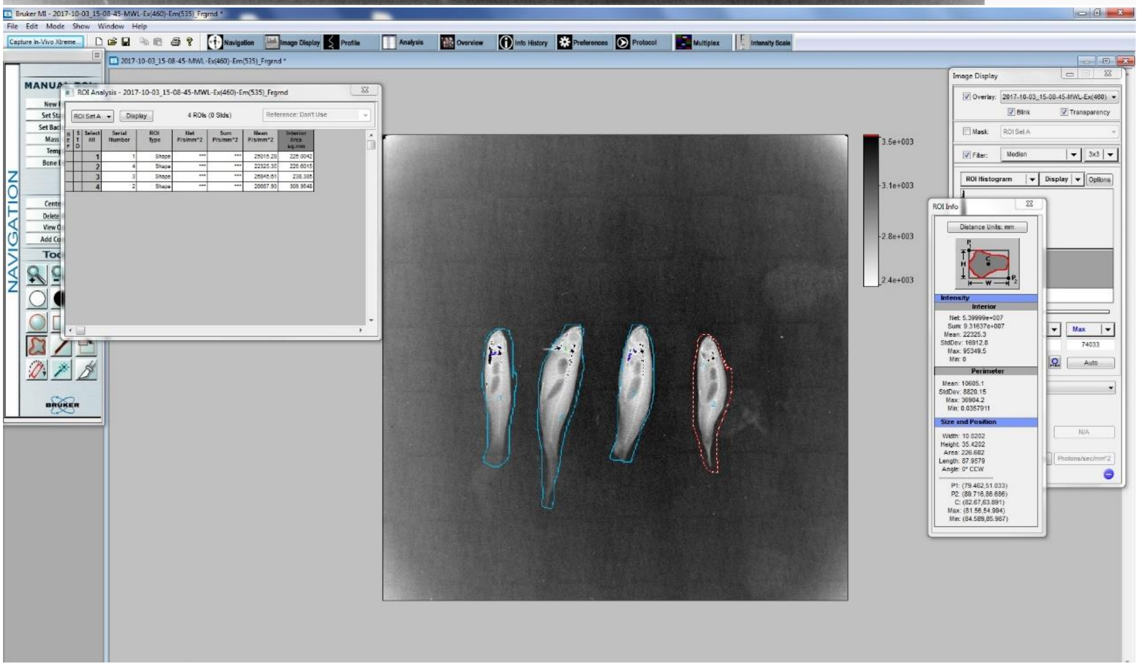

Supplementary information

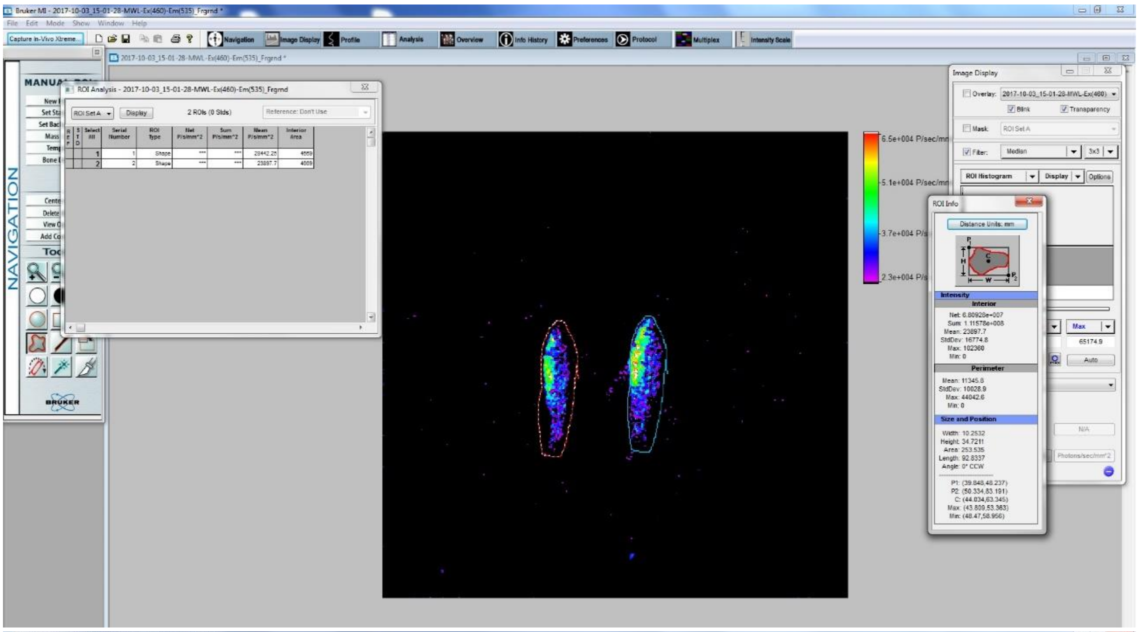

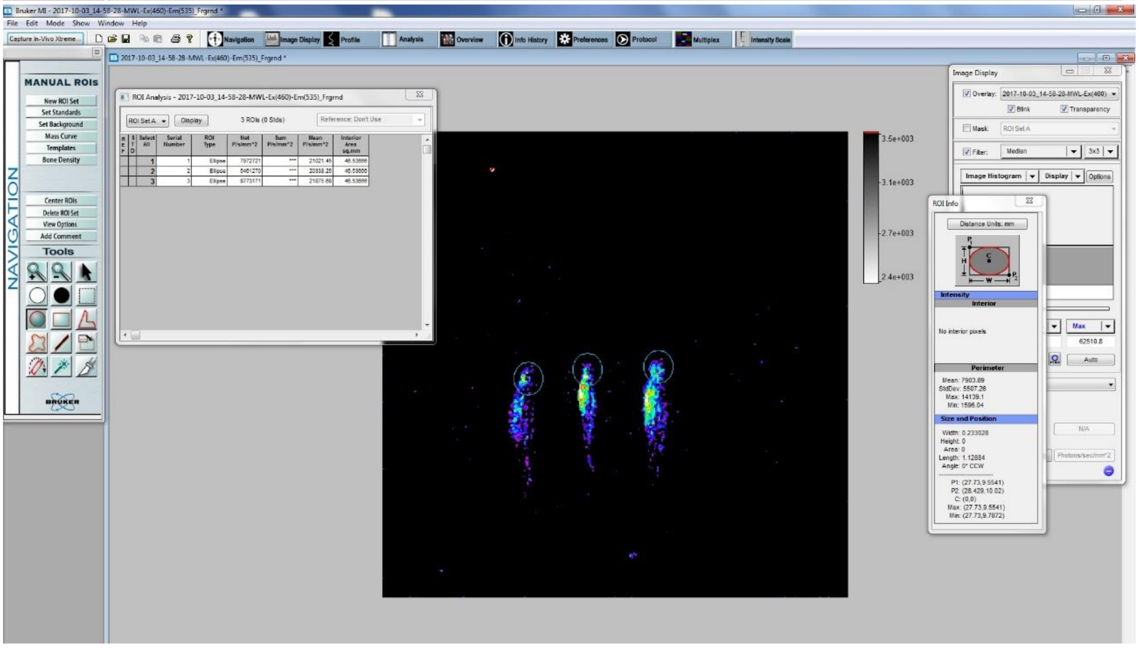

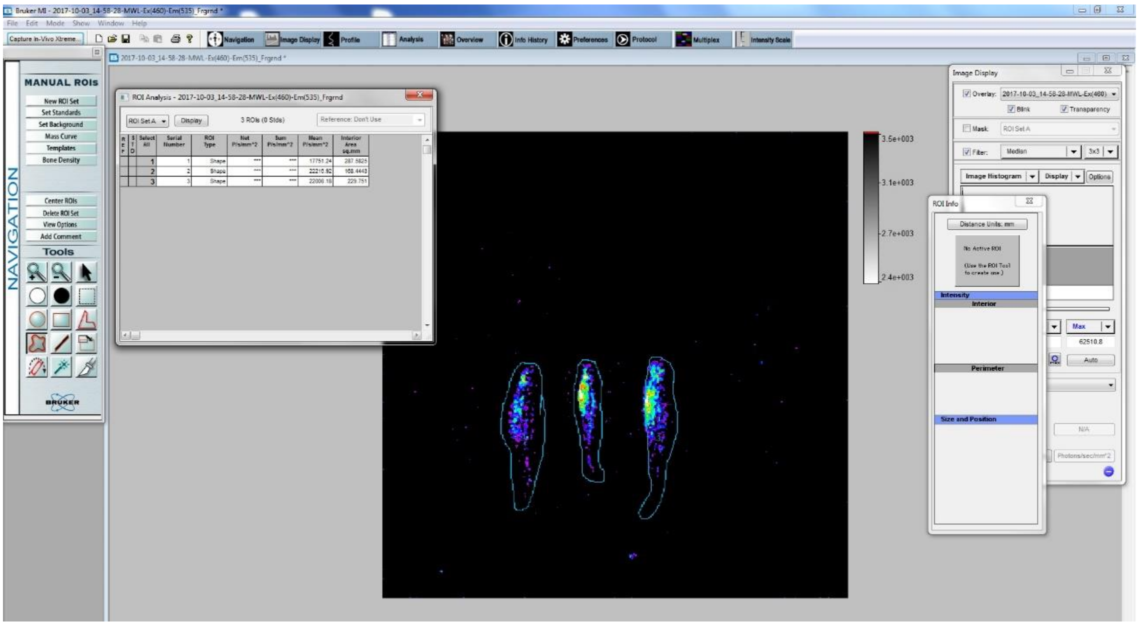

MANUAL ROIs
NAVIGATION
Tools
Image Display
ROI Info
Intensity
Interior
Perimeter
Size and Position
3.5e+003
3.1e+003
2.7e+003
2.4e+003

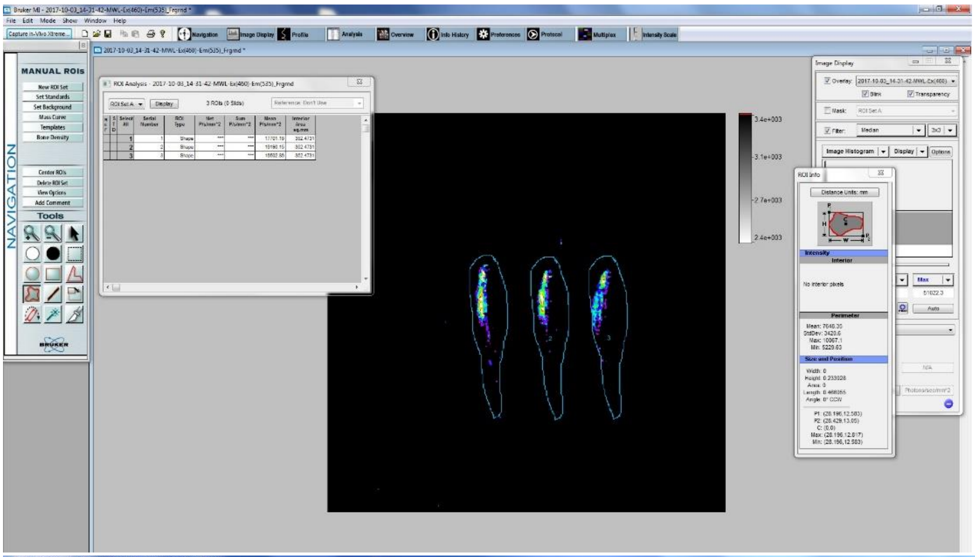
MANUAL ROIs
NAVIGATION
Tools

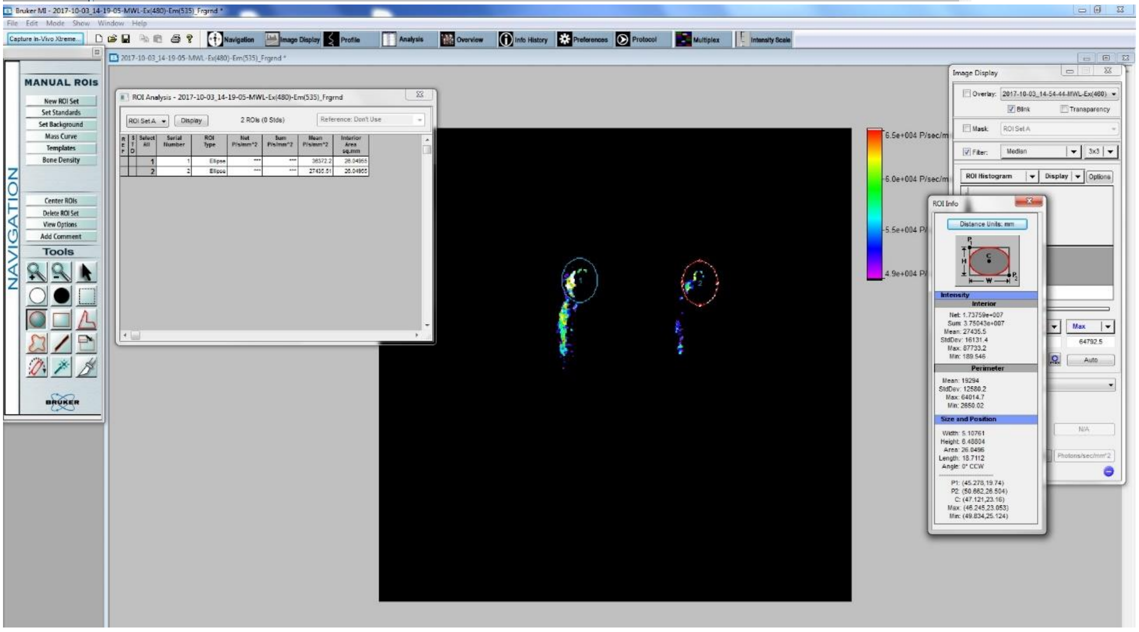
MANUAL ROIs
NAVIGATION
Tools

Supplementary information

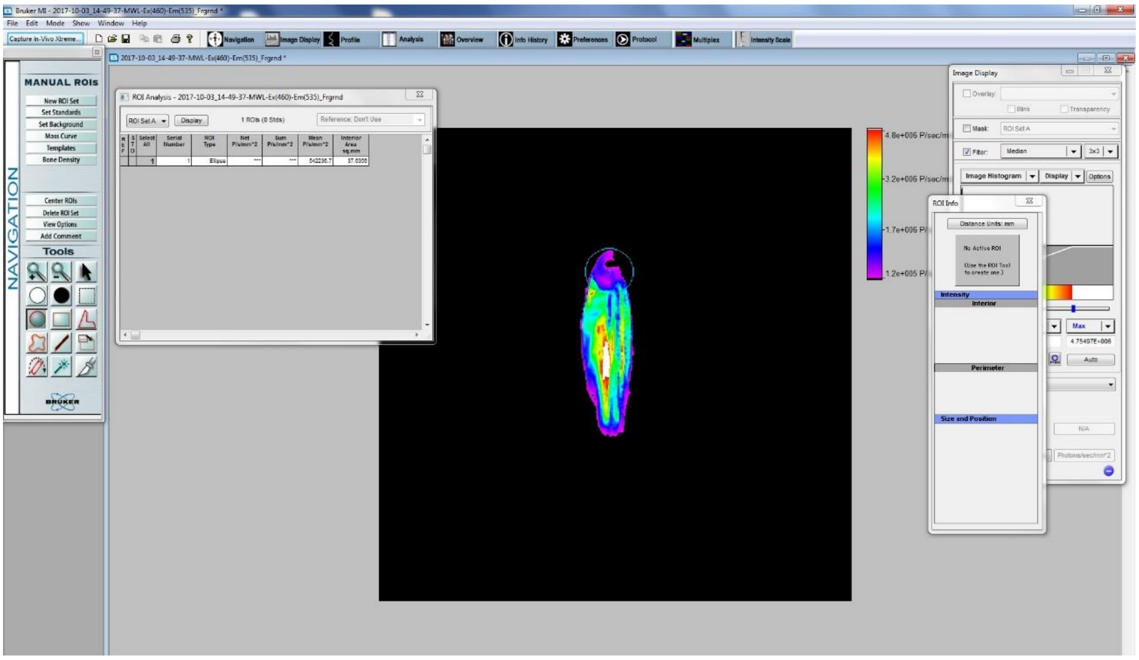

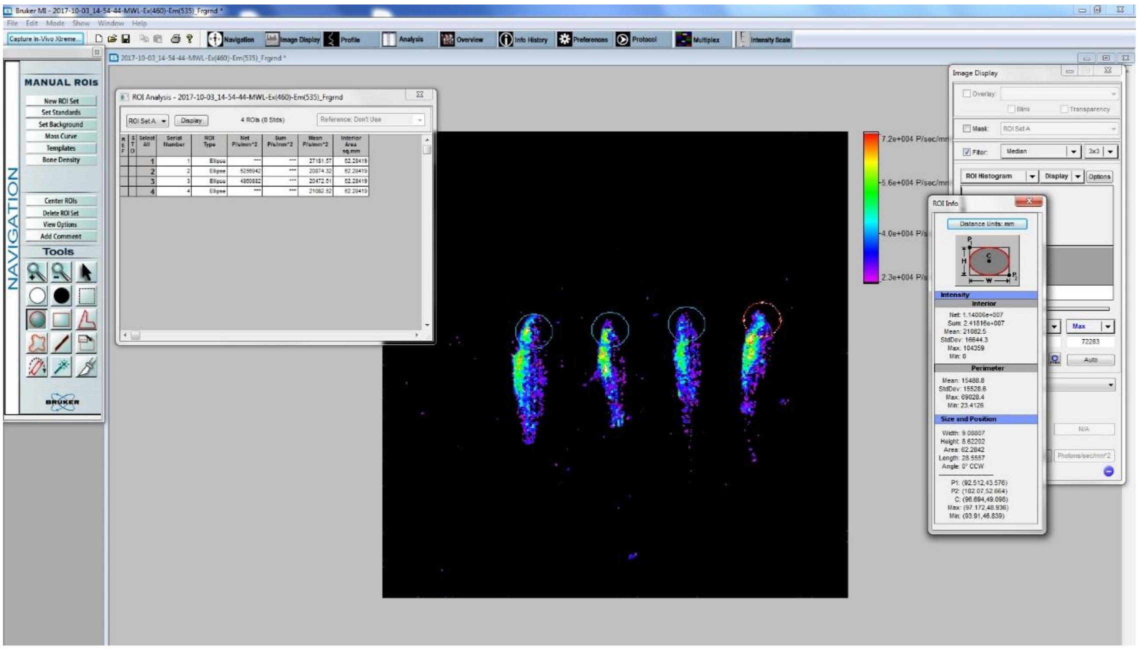

Supplementary information

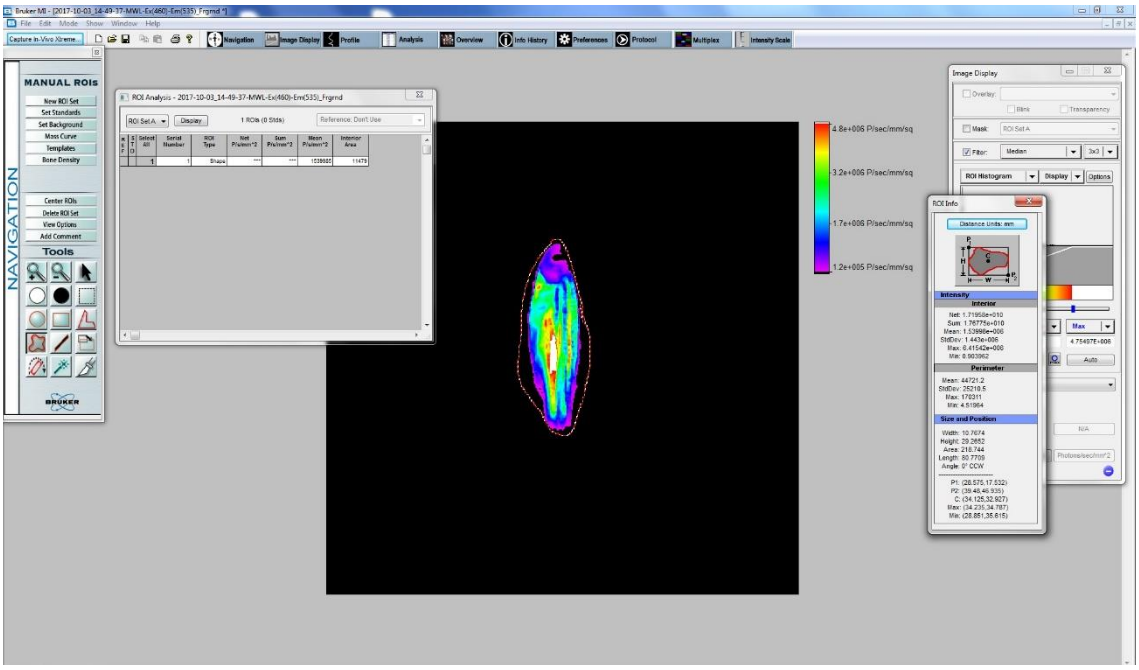

Supplementary information

Survival percentage of embryos exposed to free BENZ and encapsulated in nanocapsules.

Outcomes:	Live =0	Dead = 1											
Artemisin													
0.125 (mg/l)	Hours	0.25 (mg/l)	Hours	0.5 (mg/l)	Hours	..0 (mg/l	Hours	!.0 (mg/l	Hours	CT(+)	Hours	CT(-)	Hours
0	96	0	96	0	96	0	96	0	96	1	24	0	96
0	96	0	96	0	96	0	96	0	96	1	24	0	96
0	96	0	96	0	96	0	96	0	96	1	24	0	96
0	96	0	96	0	96	0	96	0	96	1	24	0	96
0	96	0	96	0	96	0	96	0	96	1	24	0	96
0	96	0	96	0	96	0	96	0	96	1	24	0	96
0	96	0	96	1	24	0	96	0	96	1	24	0	96
1	24	0	96	0	96	0	96	0	96	1	96	0	96
0	96	0	96	0	96	0	96	0	96	1	24	0	96
0	96	0	96	0	96	0	96	0	96	1	24	0	96
0	96	0	96	0	96	0	96	0	96	1	24	0	96
0	96	0	96	0	96	0	96	0	96	1	24	0	96
0	96	0	96	0	96	0	96	0	96	1	72	0	96
0	96	0	96	0	96	0	96	0	96	1	24	0	96
0	96	0	96	0	96	0	96	0	96	1	24	0	96
0	96	0	96	0	96	0	96	0	96	1	24	0	96
0	96	0	96	0	96	0	96	0	96	1	24	0	96
0	96	0	96	0	96	0	96	0	96	1	24	0	96
0	96	1	24	0	96	0	96	0	96	1	24	0	96
0	96	0	96			0	96	0	96	1	24	0	96
NP													
0.125 (mg/l)	Hours	0.25 (mg/l)	Hours	0.5 (mg/l)	Hours	..0 (mg/l	Hours	!.0 (mg/l	Hours	CT(+)	Hours	CT(-)	Hours
0	96	0	96	0	96	0	96	0	96	1	24	0	96
0	96	0	96	0	96	0	96	0	96	1	24	0	96
0	96	0	96	0	96	0	96	0	96	1	24	0	96
0	96	0	96	0	96	0	96	0	96	1	24	0	96
0	96	0	96	0	96	0	96	0	96	1	24	0	96
0	96	0	96	0	96	0	96	0	96	1	24	0	96
0	96	0	96	0	96	0	96	0	96	1	24	0	96
0	96	0	96	0	96	0	96	0	96	1	96	0	96
0	96	0	96	0	96	0	96	0	96	1	24	0	96
0	96	0	96	0	96	0	96	0	96	1	24	0	96
0	96	0	96	0	96	0	96	0	96	1	24	0	96
0	96	0	96	0	96	0	96	0	96	1	24	0	96
0	96	0	96	0	96	1	24	0	96	1	72	0	96
0	96	0	96	0	96	0	96	0	96	1	24	0	96
0	96	0	96	0	96	0	96	0	96	1	24	0	96
0	96	0	96	0	96	0	96	0	96	1	24	0	96
0	96	0	96	0	96	0	96	0	96	1	24	0	96
0	96	0	96	0	96	0	96	0	96	1	24	0	96
0	96	0	96	0	96	0	96	0	96	1	24	0	96
0	96	0	96	0	96	0	96	0	96	1	24	0	96
Artemisin+NP													
0.125 (mg/l)	Hours	0.25 (mg/l)	Hours	0.5 (mg/l)	Hours	..0 (mg/l	Hours	!.0 (mg/l	Hours	CT(+)	Hours	CT(-)	Hours
0	96	0	96	0	96	0	96	0	96	1	24	96	0
0	96	0	96	0	96	0	96	0	96	1	24	96	0
0	96	0	96	0	96	0	96	0	96	1	24	96	0
0	96	0	96	0	96	0	96	0	96	1	24	96	0
0	96	0	96	0	96	0	96	0	96	1	24	96	0
0	96	0	96	0	96	0	96	0	96	1	24	96	0
0	96	0	96	0	96	0	96	0	96	1	24	96	0
1	24	0	96	0	96	0	96	0	96	1	96	96	0
0	96	0	96	0	96	0	96	0	96	1	24	96	0
0	96	0	96	0	96	0	96	0	96	1	24	96	0
0	96	0	96	0	96	0	96	0	96	1	24	96	0
0	96	0	96	0	96	0	96	0	96	1	24	96	0
0	96	0	96	0	96	0	96	0	96	1	72	96	0
0	96	0	96	0	96	0	96	0	96	1	24	96	0
0	96	0	96	0	96	1	24	0	96	1	24	96	0
0	96	0	96	0	96	0	96	0	96	1	24	96	0
0	96	0	96	0	96	0	96	0	96	1	24	96	0
0	96	0	96	0	96	1	24	0	96	1	24	96	0
0	96	0	96	0	96	0	96	0	96	1	24	96	0
0	96	0	96	0	96	0	96	0	96	1	24	96	0
